# Pipe experiment elucidates biochar application depth affects nitrogen leaching under crop present condition

**DOI:** 10.1038/s41598-024-73621-3

**Published:** 2024-10-01

**Authors:** Kosuke Hamada, Satoshi Nakamura, Daichi Kuniyoshi

**Affiliations:** 1 Tropical Agriculture Research Front, Japan International Research Center for Agricultural Sciences, Ishigaki, Japan; 2 Crop, Livestock and Environmental Division, Japan International Research Center for Agricultural Sciences, Tsukuba, Japan

**Keywords:** Bagasse biochar, Soil-water stress, Groundwater pollution, Environmental load, Environmental sciences, Hydrology

## Abstract

Nitrogen leaching, resulting from the inefficient use of fertilizers, pollutes the environment, such as groundwater. Biochar can be applied to farmlands to mitigate nitrogen leaching. The effect depends on the application depth. However, the effect has not been examined under crop-farming conditions. Evaluating the interactions between biochar application depth and crop growth is indispensable for considering depth in the actual field. To address this, we conducted a pipe experiment with four treatments, no biochar (control), surface (0–5 cm), plow layer (0–30 cm), and subsurface (25–30 cm) applications, and compared the results with no-crop conditions from a previous study. Biochar application depth affected soil NO_3_^−^−N and NH_4_^+^−N absorption ability and also influenced soil-water stress conditions, affecting crop growth. Surface biochar application improved nitrogen absorption and reduced soil-water stress, improving crop growth. The NO_3_^−^−N leaching was reduced to 87.7%. Plow layer application worsened nitrogen absorption and resulted in frequent dry stress in the shallow-soil layer, preventing root growth in this layer. The NO_3_^−^−N and NH_4_^+^−N leaching increased 106.4% and 264.1%, respectively. The effects of subsurface application were similar to those in the control. Selecting an appropriate application depth can simultaneously improve crop growth and reduce nitrogen leaching.

**Supplementary Information:**

The online version contains supplementary material available at 10.1038/s41598-024-73621-3.

## Introduction

Nitrogen contamination (in the form of reactive nitrogen, comprising all nitrogen compounds except N_2_) is considered a severe environmental problem. By exceeding the Earth’s systems thresholds, nitrogen contamination puts current and future living organisms at risk^1^. Since the development of the Haber–Bosch process, massive amounts of nitrogen have been applied to farmlands as fertilizer, increasing crop yields^2^. However, this has led to the inefficient use of nitrogen, causing its loss to the environment via volatilization and leaching, harming both the environment and human health^3^. Addressing this problem in agriculture holds promise for solving these issues, because agricultural use accounts for a large part of the nitrogen load in the environment^4^. 50% of the nitrogen applied in agricultural production in the European Union (EU) is not taken up by crops but is released into the environment^5,6^.

The primary component of agricultural nitrogen fertilizers is ammonium nitrogen (NH_4_^+^−N), which is converted into nitrate nitrogen (NO_3_^−^−N) via nitrification. Because most soils contain more negatively charged sites than positively charged sites, NO_3_^−^−N leaches more readily than NH_4_^+^−N and pollutes water bodies such as groundwater. This problem is severe on subtropical/tropical islands; first because the earth on these islands primarily comprises limestone, which is highly permeable to water^7^, and second because sugarcane, the primary industry of these islands, has low fertilizer-use efficiency^8^. NO_3_^−^−N leaching affects drinking water quality, putting humans at risk^3^, and degrades coral, which plays a significant role in maintaining regional biodiversity^9,10^. Therefore, mitigation of leaching is urgently required on these islands.

Biochar, which is made from biomass in an oxygen-limited environment with low temperature (< 800 °C), is used in farmlands for carbon sequestration^11^ and a promising measure to mitigate NO_3_^−^−N leaching. Biochar can, for instance, physically trap NO_3_^−^−N and NH_4_^+^−N and chemically bind them via functional groups on the biochar surface^12,13^. This improves the physical and chemical properties of the soil^14–16^ and nitrogen use efficiency (NUE)^13^ by the slow release of NO_3_^−^−N. Moreover, biochar positively affects crop yield; for example, Liu et al.^17^ reported an increase in rice yield and NUE after biochar application. The properties and effects of biochar differ depending on the feedstock, pyrolysis temperature, and other factors^18,19^. Many studies have evaluated the effects of biochar application rates^20^ and suggested their cost-effectiveness^21^. Biochar application depth also affects soil-water movements such as evaporation and infiltration, as well as NO_3_^−^−N leaching^22,23^. Hamada et al.^23^ conducted a pipe experiment without crops, using the same amount of bagasse biochar in each pipe, evaluating the effects of surface (0–5 cm), plow layer (0–30 cm), and subsurface (25–30 cm) biochar applications. Their results revealed that the water budget (soil-water evaporation, amount of water in the pipe, and drainage) and consequently NO_3_^−^−N leaching varied with biochar application depth. The study indicated that selecting an appropriate application improves biochar application efficiency.

Biochar application alters crop-root development (distribution and length) and total biomass yield^24^. Moreover, the uptake of water and nitrogen by crop root varies depending on soil-water conditions^25,26^. Therefore, changes in soil water conditions induced by biochar application depth would alter crop-root development and water and nitrogen intake, and different crop conditions would enhance/mitigate the effect of biochar application depth. However, no study has evaluated the effect under crop-farming conditions. Hence, it is necessary to evaluate the effect of biochar application depth under crop-present conditions and understand the mechanism driving this effect, as this is indispensable to implementing biochar application in actual fields.

We aimed to evaluate the interactions between biochar application depths and crop growth and their effect on nitrogen leaching and understand the mechanism driving this effect. We conducted a pipe experiment using upland rice under four experimental conditions: no biochar, surface, plow layer, and subsurface applications. By comparing the findings with those of a previous study^23^, we attempted to elucidate the mechanisms by distinguishing the effect of application depth and differences in crop growth. We hypothesized that (a) the difference in water budget, associated with biochar application depth, would affect soil-water stress conditions, water and nitrogen uptake by crop, and consequently, crop growth; and (b) the trend in NO_3_^−^−N leaching under crop growth would differ from that observed in the absence of crop growth reported by Hamada et al.^23^

## Materials and methods

### Pipe experiment

The pipe experiment (**Supplementary Fig. S1**) was conducted from January 12, 2022, to April 13, 2022 (91 days) in a glass room at 23 °C and 70% average humidity at the Tropical Agriculture Research Front (TARF), Japan International Research Center for Agricultural Sciences (24°22ʹ43ʺ N; 124°11ʹ43ʺ E). The experiment was designed based on a previous study^23^. Four treatments were used: no biochar, surface (0–5 cm; treatment A), plow layer (0–30 cm; treatment B), and subsurface (25–30 cm; treatment C) applications. The applied biochar was mixed with soil in each treatment. We used commercially sold biochar (Fujimibussan Co., Ltd., Shizuoka, Japan), made of sugarcane bagasse with a pyrolysis temperature of 800 °C. The applied soil is classified as Dystric Cambisol according to the World Reference Base for Soil Resources^27^. The biochar was 2 mm-sieved, and the soil was 6 mm-sieved. The chemical properties of the soil and biochar are illustrated in **Supplementary Tables S1 and S2**.

We used vinyl chloride pipes with a diameter of 20 cm and height of 100 cm. Each pipe had a valve at the bottom for drainage control. We applied four treatments with five replicates of each. The soil bulk density was set to 1.25 g cm^− 3^ in each pipe. The amount of applied biochar was the same among treatments A, B, and C (10 t ha^− 1^). Biochar content rates (expressed as weight ratios) in the biochar amendment layer were 1.57% for treatments A and C, and 0.26% for treatment B. To pack soil as uniformly as possible in the pipes, we added soil while tapping constantly and gently. To prevent preferential flow, we saturated the soil before the experiment. The valves were fully opened after the saturation and during the experiment. Surface irrigation was conducted every two or three days using tap water. The amount of water for each irrigation was 17 mm (550 mL pipe^− 1^). We determined the amount and times of irrigation based on precipitation data from the local meteorological observatory (24°20ʹ N; 124°09ʹ E) managed by the Japan Meteorological Agency (located 5.6 km southwest of the experiment location). We supplied fertilizer powder on three occasions: KH_2_PO_4_ and (NH_4_)_2_SO_4_ on January 12, 2022, and (NH_4_)_2_SO_4_ on February 14 and March 14, 2022. Each fertilizer was applied to the soil surface before irrigation. When irrigating, we watered carefully to dissolve all the fertilizer. The total amount of applied nitrogen was set to 600 kg N ha^− 1^ to ensure NO_3_^−^−N leaching.

Upland rice (NERICA 4) was planted at the center of each pipe. The NERICA varieties were bred by AfricaRice using an interspecific cross of Asian (*Oryza sativa*) and African (*Oryza glaberrima*) rice^28,29^), registered as a variety; therefore, no permission was necessary for research uses. Before the experiment, upland rice seeds were immersed in water for a day and then incubated in a humid box under dark conditions for 1 week at 25 °C. Three germinated seeds per pipe were carefully planted around the center of the pipe on January 11, 2022. Two plantlets were removed from each pipe on February 7, 2022.

### Sampling and measurement

Soil moisture sensors (TEROS12; METER Group, Pullman, WA, USA) and samplers (DIK-301B; Daiki Rika Kogyo Co., Kounosu, Japan) were installed in all pipes (five replicates per treatment) at depths of 10, 20, 35, and 80 cm. Drainage was collected from all pipes before each irrigation, and the weight of the drainage was measured. The concentration of NO_3_^−^−N and NH_4_^+^−N in soil solution and drainage was measured using the Auto Analyzer III (BL TEC K. K., Tokyo, Japan).

The height of the crops was measured as the length between surface of the ground and the tip of the topmost fully expanded leaf. Crop height per pipe was measured every 2 weeks for all pipes. After the pipe experiment, we counted the number of tillers for each crop and collected the aboveground parts (five replicates). The roots were collected from three pipes per treatment. Sampling depths were 0–10, 10–20, 20–30, 30–40, 40–65, and 65–95 cm. Using the method proposed in Murakami et al.^30^, the roots were separated from the soil and root length density was determined in each soil layer. Aboveground parts and roots were dried for 1 week at 70 °C, then dry matter weight and total nitrogen (TN) were measured using an NC analyzer (NC 220 F; Sumika Chemical Analysis Service, Ltd., Tokyo, Japan).Undisturbed and disturbed soils were collected at depths of 5, 30, and 55 cm from three pipes per treatment to evaluate the soil water retention curve and saturated soil hydraulic conductivity, as well as NO_3_^−^−N, NH_4_^+^−N and TN content.

NUE was estimated using crop body TN, fertilizer nitrogen applied, and NO_3_^−^−N and NH_4_^+^−N amount in the soil. Soil NO_3_^−^−N and NH_4_^+^−N were obtained by multiplying the weight of the soil column in the pipes by the NO_3_^−^−N and NH_4_^+^−N content of the initial soil. The NO_3_^−^−N and NH_4_^+^−N contents in the no-biochar treatment and biochar-amended soils under treatments A and C, and treatment B, respectively, were considered in the calculation.

Dunnett’s test was conducted to evaluate the significance of differences from the control with a significance threshold of *p* < 0.05 and *p* < 0.01. As per the methods of Hamada et al.^23^, the maximum and minimum values for drainage, NO_3_^−^−N and NH_4_^+^−N leaching, crop height, and TN in the aboveground parts were first removed from the data before analysis, and data from three replicates were used for each analysis. It is worth noting that the test to the root and volumetric water content could not be conducted because there were only two replicates.

### Conversion of volumetric water content into matric potential head and estimation of soil-water stress

We considered soil-water stress conditions (dry and wet) because these conditions reduce water and nitrogen uptake by the plant^25,26^, thus reducing crop growth^31,32^. To evaluate soil-water stress, which also affect the root distribution, we converted *θ* into matric potential head (*h*) using the van Genuchten model^33^.

1 $$\:\theta\:\left(h\right)={\theta\:}_{res}+\left({\theta\:}_{sat}-{\theta\:}_{res}\right){\left(1+{\left|\alpha\:h\right|}^{n}\right)}^{-m}$$

where *θ*_*res*_ and *θ*_*sat*_ denote the residual and saturated volumetric water content (cm^3^ cm^− 3^), respectively, and *α* (cm^− 1^), *n* (–), *m* (= 1 − 1/*n*), and *λ* (= 0.5) are the empirical shape factors. The empirical shape factors were first obtained by fitting the model to each measured soil-water retention curve, then used to convert the measured *θ* to *h*. The curves were applied at a depth of 5 cm to *θ* at a depth of 10 cm, at a depth of 30 cm to *θ* at depths of 20 and 35 cm, and at a depth of 55 cm to *θ* at a depth of 80 cm. Wet stress was considered to occur at *h* > − 10 cm^34^, and dry stress at *h* ≤ − 1.0 × 10^3.0^ cm, based on Miyazaki and Nishimura^35^. We did not consider *h* < − 1.0 × 10^4.2^ cm (the permanent wilting point) because crops cannot absorb soil water after that point^36^. Water uptake was considered to decrease at values above the wet threshold and below the dry threshold, based on Feddes et al.^37^ This assumption is conventionally applied^25^.

### Nutrient budget and water balance

The nutrient budget was estimated by the following procedures: (i) logarithmic regression was conducted to the observed TN, which was collected from three depths (5, 30, and 55 cm), to obtain the distribution of TN with depth. (ii) we assumed that because of surface irrigation, the soil bulk density was changed with depth; we thus changed the soil bulk density linearly from 1.15 to 1.35 g cm^− 3^ with depth. (iii) the amount of TN was calculated in every 1–2 cm soil column and obtained for the whole soil column. The soil TN was converted into soil NO_3_^−^−N and NH_4_^+^−N by logarithmic regression of NO_3_^−^−N/TN and NH_4_^+^−N/TN with depth using the observed contents of NO_3_^−^−N and NH_4_^+^−N in soil. The amounts of NO_3_^−^−N and NH_4_^+^−N in the soil column were estimated by multiplying the amount of TN with NO_3_^−^−N/TN and NH_4_^+^−N/TN in each depth.

Water balance was calculated the same method as Hamada et al.^23^ The average *θ*, average cumulative amount of drainage, and amount of irrigation were used. To estimate the amount of water in each pipe, we assumed that the sensors at depths of 10, 20, 35, and 80 cm represent soil layers with heights of 15, 15, 20, and 45 cm, respectively. The sensor values were multiplied by the soil layer volumes. Evapotranspiration was calculated by subtracting the amount of water in pipe and drainage from the amount of irrigation.

## Results

### Crop height

Crop height was greater in treatment A than in the control, although non-significantly (Table 1): On February 21, crop height was only 0.4 cm higher in treatment A than in the control, with larger differences on March 14 and 28 (3.8 and 3.9 cm, respectively), and a smaller difference on the final day (1.8 cm). Under treatment B, crop height was higher than that in the control until March 14, when the largest difference was observed (2.9 cm); on March 28, however, crop height was larger in the control; and on the final day, the height was nearly the same in these groups. Under treatment C, from January 27 to February 21, crop height was slightly higher than that in the control (by 1–0.8 cm), and on March 14, it was 1.9 cm higher than that in the control; however, after March 14, it was higher in the control, reaching 2.0 cm higher on the final day. The final-day crop heights were the greatest in treatment A; less and equal in the control and treatment B, respectively; and lowest in treatment C. Tiller number was the highest in treatment A but the same for the other treatments (Table 1).

**Table 1:** Temporal changes in crop height and tiller number.

Date	Control	A	B	C
27 January 2022	14.8 ± 0.09	16.5 ± 0.20 ^ns^	16.3 ± 0.33 ^ns^	14.9 ± 0.43 ^ns^
7 February 2022	31.5 ± 0.02	33.7 ± 0.59 ^ns^	33.4 ± 0.52 ^ns^	31.9 ± 0.58 ^ns^
21 February 2022	47.8 ± 0.11	48.2 ± 0.66 ^ns^	49.1 ± 0.69 ^ns^	48.6 ± 0.44 ^ns^
14 March 2022	79.0 ± 1.56	82.8 ± 0.65 ^ns^	81.9 ± 0.86 ^ns^	80.9 ± 0.89 ^ns^
28 March 2022	86.7 ± 0.54	90.6 ± 1.07 ^ns^	85.4 ± 1.04 ^ns^	85.0 ± 0.58 ^ns^
13 April 2022	107.3 ± 0.66	109.1 ± 1.05 ^ns^	107.2 ± 0.84 ^ns^	105.3 ± 0.11 ^ns^
Tiller number	7 ± 0.4	8 ± 0.4 ^ns^	7 ± 0.6 ^ns^	7 ± 0.2 ^ns^

Unit of crop height: cm. Control: no biochar; A: surface application; B: plow layer application; C: subsurface application. The average values of three replicates are displayed. ns indicates non-significant difference compared with the control. ± represents standard error.

### Nitrogen intake

**Fig. 1:**
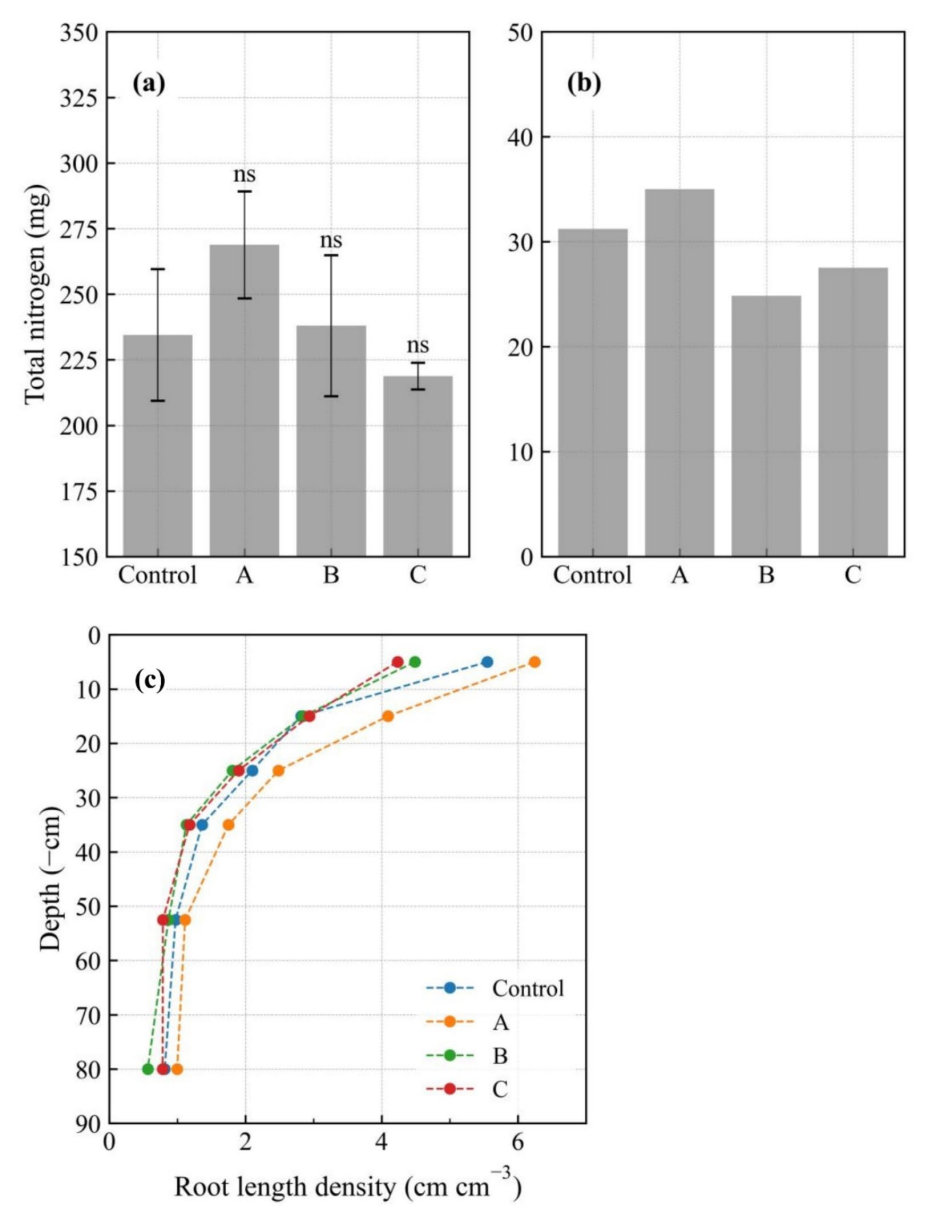
Total nitrogen (**a**) in aboveground, (**b**) in the roots, and (**c**) distribution of root length density with depth. Control: no biochar; A: surface application; B: plow layer application; C: subsurface application. The average values are based on three replicates for the aboveground parts and two replicates for the roots. The letters ns indicate a non-significant difference compared with the control. Error bar indicates the standard error (*n* = 3).

The differences among the treatments in aboveground TN were the same as those for crop height (Fig. 1a). In treatment A, final-day aboveground TN was 114.7% that of the control; for treatments B and C, these factors were 101.5% and 93.3%, respectively. Aboveground TN was the highest in treatment A, as a result of both increased crop height and tiller number. Relative to the control, root TN was 112.1%, 79.6%, and 88.1% in treatments A, B, and C, respectively (Fig. 1b), in contrast to trends observed for crop height and aboveground TN. The average crop TN (aboveground + root) was 265.7, 363.8, 262.8, and 246.3 mg for the control and treatments A, B, and C, respectively. NUE was 12.4%, 14.1%, 12.1%, and 11.4% for the control and treatments A, B, and C, respectively, which were similar to the NUE reported by Liu et al.^17^

### Distribution of root length density with depth

The total root lengths were 52,473, 63,915, 44,428, and 45,752 cm (100.0%, 121.8%, 84.7%, and 87.2%) in the control and treatments A, B, and C, respectively. Similarly, the treatments differed in terms of root distribution with depth (Fig. 1c). At all depths, root length density was higher in treatment A than in the control, at 112.5–145.3% of the control value. The largest difference (1.3 cm cm^− 3^) was found at a depth of 10–20 cm. In treatments B and C, root length density tended to be lower than that in the control at all depths except 10–20 cm; at a depth of 0–10 cm, relative to the control, root length density was 1.1 cm cm^− 3^ smaller (at 80.9%) in treatment B and 1.3 cm cm^− 3^ smaller (at 76.3%) in treatment C, which exhibited the largest difference. In treatment B, root length density was the lowest in the deepest soil layer, at 69.4% that of the control.

### Soil water and nitrogen movement

#### Temporal changes in soil-solution NH_4_^+^−N and NO_3_^−^−N

**Fig. 2:**
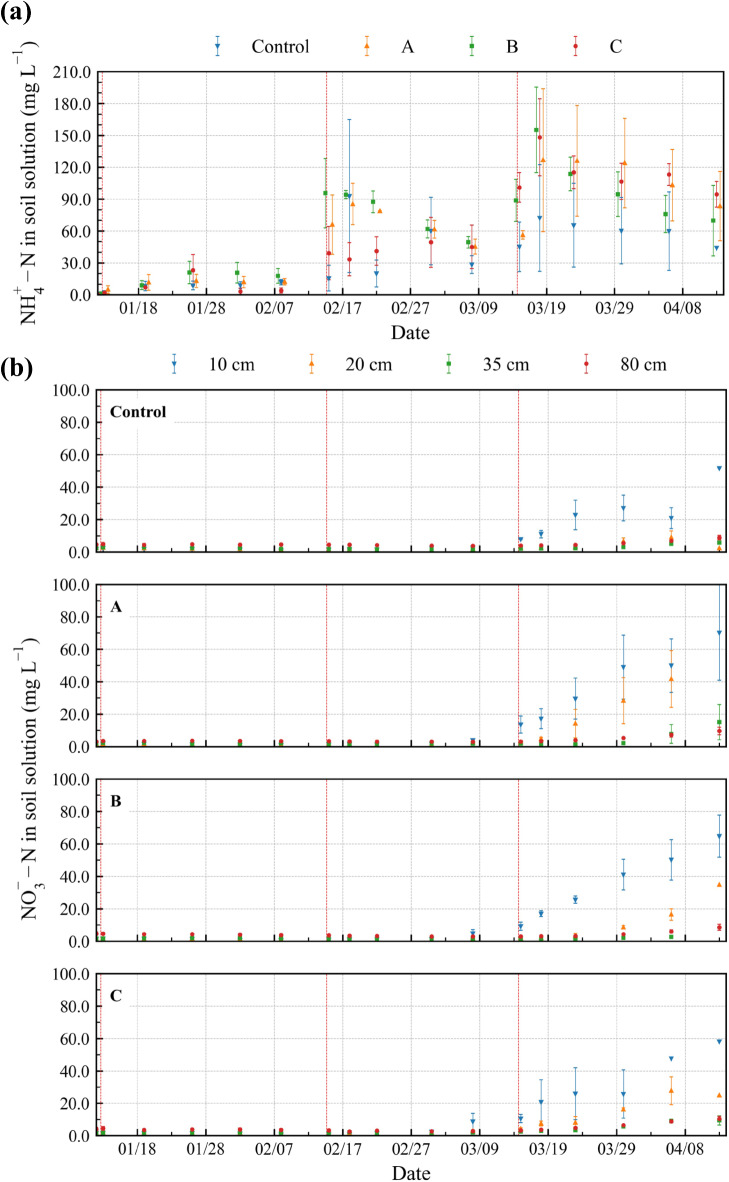
Temporal changes in the concentration of (**a**) dissolved NH_4_^+^−N at a depth of 10 cm and (**b**) dissolved NO_3_^−^−N. Control: no biochar; A: surface application; B: plow layer application; C: subsurface application. The vertical red dashed lines indicate fertilizer application. The average values of three replicates are shown. Error bar indicates the standard error (*n* = 3).

Under all treatments at depths deeper than 20 cm, the concentration of dissolved NH_4_^+^−N was < 2.5 mg L^–1^, varying little among the treatments (data not shown). Variability in NH_4_^+^−N concentration among treatments was high at a depth of 10 cm (Fig. 2a). NH_4_^+^−N concentration increased with the number of fertilizer applications: for all treatments, it was < 30 mg L^–1^ after one application, reaching 5–100 and 45–160 mg L^–1^ after two and three applications, respectively. With an increase in the number of applications, the differences in NH_4_^+^−N concentration between the treatments increased; after three applications, it was higher in treatments A–C than in the control.

Before application three, NO_3_^−^−N concentration was < 5.0 mg L^–1^ in all treatments, differing little among the treatments (Fig. 2b). NO_3_^−^−N concentration increased after application three, as did the difference among the treatments. In the control, NO_3_^−^−N concentration increased notably with fertilizer application only at a depth of 10 cm; at the other depths, it remained < 10 mg L^–1^. In treatments A and B, NO_3_^−^−N concentration increased with fertilizer application at depths of 10 and 20 cm. At a depth of 20 cm, it increased faster in treatment A than in treatment B. Treatment C exhibited the smallest increase in NO_3_^−^−N concentration at a depth of 10 cm, with an increase at a depth of 20 cm.

#### Soil NO_3_^−^−N, NH_4_^+^−N and TN content

Variability in soil NO_3_^−^−N and NH_4_^+^−N content was the highest in the shallowest soil layer (Table 2). On the final day, the NO_3_^−^−N content in the control and treatments A and C in the shallowest soil layer was double its initial value, whereas in treatment B, it was almost the same as the initial value. At depths deeper than 30 cm, NO_3_^−^−N content after the experiment was lower than the initial value in all treatments, reaching ca. 50%.

**Table 2:** NO_3_^−^−N, NH_4_^+^−N and TN contents in the soil.

(a) NO_3_^−^–N (mg kg^–1^)								
Depth (cm)	Control		A		B		C	
	Initial	Last	Initial	Last	Initial	Last	Initial	Last
5	1.29	2.85 ± 0.37	1.27	3.47 ± 1.30^ ns^	1.23	1.42 ± 0.63^ ns^	1.29	2.71 ± 0.81^ ns^
30	1.29	0.93 ± 0.38	1.29	0.74 ± 0.25^ ns^	1.23	0.63 ± 0.07^ ns^	1.27	0.65 ± 0.25^ ns^
55	1.29	0.63 ± 0.02	1.29	0.64 ± 0.06^ ns^	1.29	0.71 ± 0.16^ ns^	1.29	0.60 ± 0.01^ ns^

(b) NH_4_^+^–N (mg kg^–1^)								
Depth (cm)	Control		A		B		C	
	Initial	Last	Initial	Last	Initial	Last	Initial	Last
5	5.81	21.98 ± 8.47	6.80	16.71 ± 6.23^ ns^	7.25	22.02 ± 2.40^ ns^	5.81	26.20 ± 9.07^ ns^
30	5.81	1.27 ± 0.25	5.81	1.56 ± 0.04^ ns^	7.25	2.76 ± 0.74^ ns^	6.80	1.03 ± 0.05^ ns^
55	5.81	1.10 ± 0.09^ s^	5.81	1.67 ± 0.10*	5.81	1.71 ± 0.10*	5.81	1.26 ± 0.16^ ns^

(c) TN (g kg^–1^)								
Depth (cm)	Control		A		B		C	
	Initial	Last	Initial	Last	Initial	Last	Initial	Last
5	0.65	1.02 ± 0.09	0.63	0.84 ± 0.04^ ns^	0.63	0.90 ± 0.03^ ns^	0.65	0.89 ± 0.03^ ns^
30	0.65	0.74 ± 0.02	0.65	0.72 ± 0.03^ ns^	0.63	0.75 ± 0.02^ ns^	0.63	0.76 ± 0.02^ ns^
55	0.65	0.76 ± 0.09	0.65	0.72 ± 0.01^ ns^	0.65	0.72 ± 0.03^ ns^	0.65	0.66 ± 0.05^ ns^

Control: no biochar; A: surface application; B: plow layer application; C: subsurface application. Values represent the means of three replicates. The initial and final values refer to the values before the experiment and the conditions on the last day, respectively. ± represents standard error. * and ns indicate significant difference (*p* < 0.05) and non-significant difference compared with the control, respectively.

Soil NH_4_^+^−N showed the same variability as soil NO_3_^−^−N. The final NH_4_^+^−N content in the shallowest soil layer was 2.5–4.5 times higher than the initial value. Soil NH_4_^+^−N content was highest in treatment C and lower in the control, followed by treatment B, and was lowest in treatment A. The increase in soil NH_4_^+^−N was higher than that in soil NO_3_^−^−N because soil can hold cations more easily than anions. By contrast, at depths deeper than 30 cm, the final-day values were 0.2–0.4 times the initial values. The fact that NH_4_^+^−N is held by the soil more strongly than NO_3_^−^−N explains why it did not migrate to the deeper soil as much as NO_3_^−^−N. This difference contributes to the smaller final/initial ratio of NH_4_^+^−N than of NO_3_^−^−N in the deeper soil layer.

Soil TN content was the highest in the shallowest soil layer and decreased with depth. However, unlike the NO_3_^−^−N and NH_4_^+^−N content, TN content in soil increased at all depths, indicating immobilization.

#### Temporal changes in volumetric water content

**Fig. 3:**
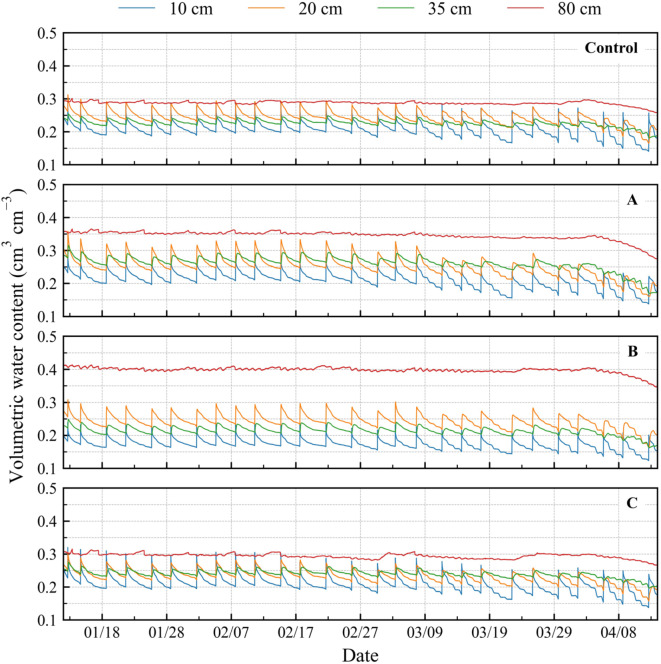
Temporal changes in the volumetric water content. Control: no biochar; A: surface application; B: plow layer application; C: subsurface application. The results at each depth were obtained by averaging the calibrated sensor data (*n* = 2).

Before March 10, 2022, the changes in *θ* over time were similar at all depths (Fig. 3). By contrast, after March 10, the minimum values of *θ* gradually decreased at depths of 10–30 cm. At a depth of 80 cm, *θ* was low after April 1, 2022. These reductions indicated root growth and increased water uptake. The treatments exhibited different trends *θ* with depth. Relative to the control, *θ* at a depth of 10 cm was the same in treatment A, while it was higher in treatment A at depths of 20 and 35 cm. In treatment B, *θ* at a depth of 10 cm was smaller than that in the control, whereas it was the same as in the control at depths of 20 and 35 cm. In treatment C, *θ* was almost the same as that in the control.

Soil physical properties (the soil water retention curve and saturated soil hydraulic conductivity) showed no significant differences among the treatments (**Supplementary Fig. S2 and Supplementary Table S3**).

### Matric potential head

**Fig. 4:**
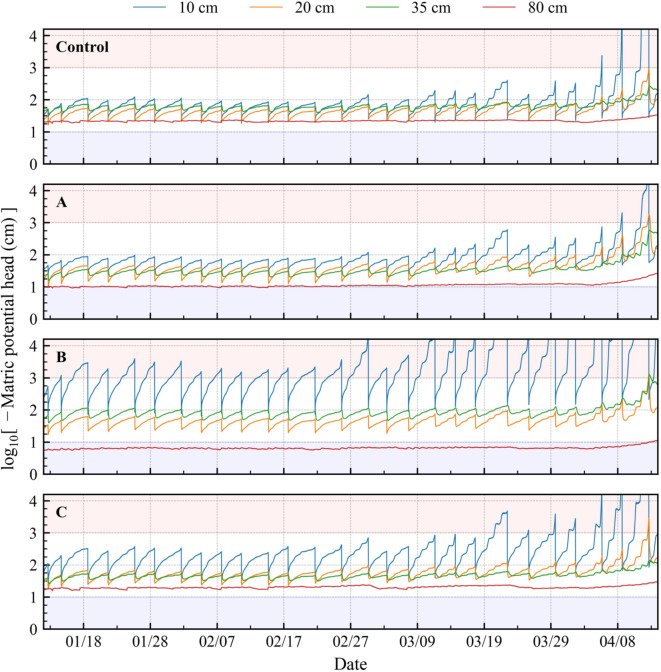
Temporal change in matric potential head at each depth. Control: no biochar; A: surface application; B: plow layer application; C: subsurface application. The matric potential head was converted from the volumetric water content (measured using TEROS12 soil moisture sensors) using soil water retention curves. The curves were obtained using the van Genuchten equation and the measured curve. The red and blue areas indicate the dry and wet stress ranges, respectively.

The matric potential head, *h*, converted from the measured *θ* (Fig. 3), exhibited differences among the treatments (Fig. 4). The average soil water retention curve was applied at a depth of 30 cm (averaged among all the treatments) to *θ* at depths of 20 and 35 cm, and the average curves at a depth of 55 cm (averaged among all the treatments) to *θ* at a depth of 80 cm, because the curves in each depth were almost identical. The van Genuchten parameters and applied soil water retention curves are presented in **Supplementary Table S4** and **Supplementary Fig. S2**.

Among all treatments, *h* at a depth of 10 cm decreased after March 18, 2022. In treatment A, *h* before March 18 was primarily between − 10 and − 100 cm, causing no water stress for the crop. Treatment A showed the lowest frequency of *h* < − 1.0 × 10^3.0^ cm, causing dry stress. Treatments B and C exhibited lower *h* at a depth of 10 cm than the control, and *h* tended to be lower in treatment B than in treatment C. In treatment B, *h* was less than − 1.0 × 10^4.2^ cm (the permanent wilting point) more frequently than that in the other treatments after March 11, 2022, followed by treatment C. At a depth of 80 cm, the *h* value of treatment B tended to be higher than − 10 cm, indicating that treatment B resulted in wet stress at this depth.

### Drainage and leaching from pipes

**Fig. 5:**
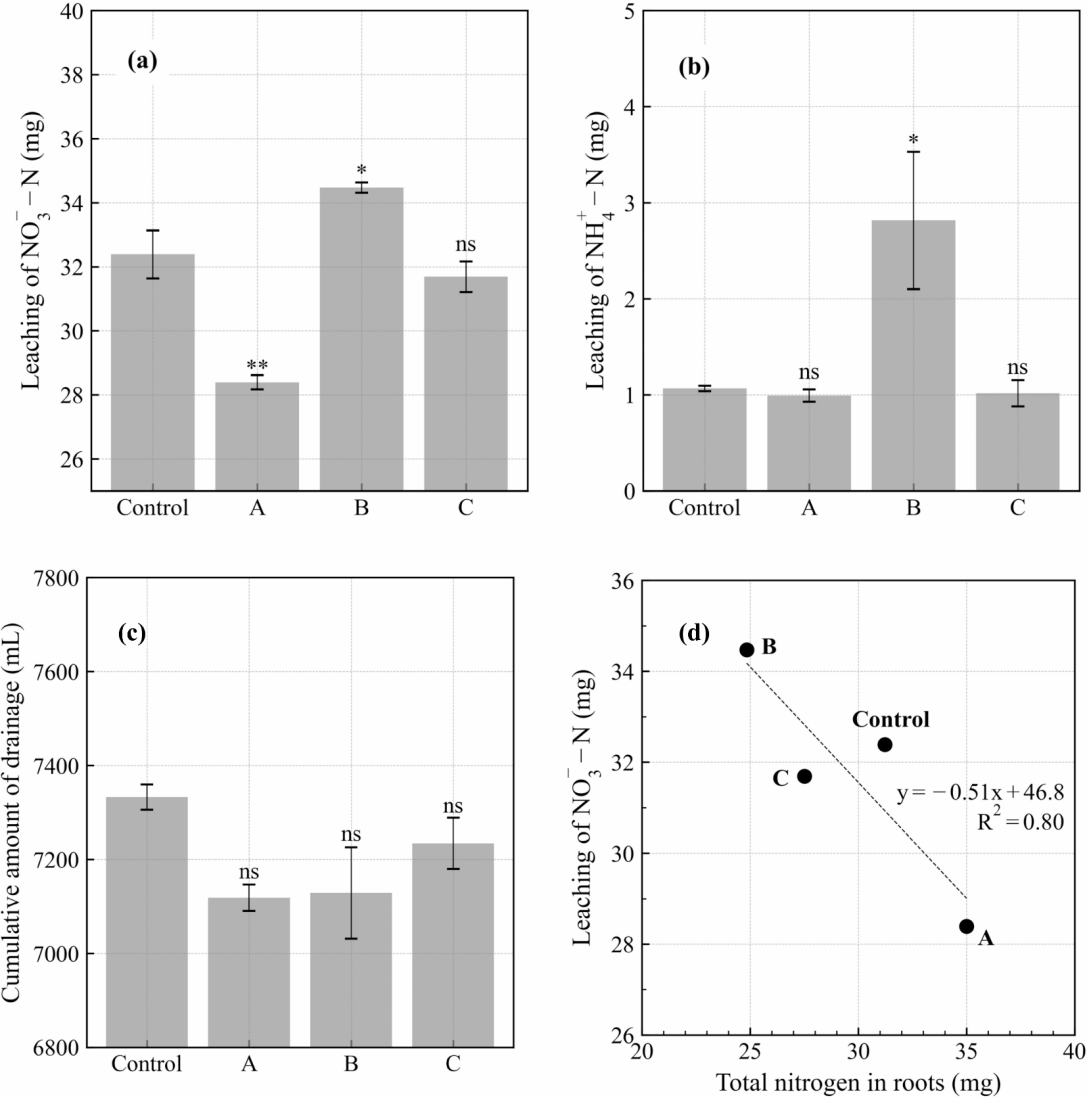
Cumulative (**a**) leaching of NO_3_^−^−N and (**b**) NH_4_^+^−N (**c**) drainage in the experiment, and (**d**) relationship between NO_3_^−^−N leaching and total nitrogen in roots. Control: no biochar; A: surface application; B: plow layer application; C: subsurface application. **, * and ns indicate a significant difference (*p* < 0.01 and *p* < 0.05) and a non-significant difference compared with the control. Error bars indicate the standard error (*n* = 3).

NO_3_^−^−N and NH_4_^+^−N leaching differed among the treatments (Fig. 5a and b). Relative to the control, cumulative NO_3_^−^−N leaching was significantly smaller in treatment A, at 87.7% (*p* < 0.01), significantly larger in treatment B, at 106.4% (*p* < 0.05), and the same in treatment C, at 97.8%. Cumulative NH_4_^+^−N leaching exhibited different variability, being the same in treatments A, C, and the control and 2.6 times greater in treatment B than in the control.

Drainage exhibited different variability to nitrogen leaching, although with no significant differences among treatments (Fig. 5c). Relative to the control, drainage was 97.1%, 97.2%, and 98.7% in treatments A, B, and C, respectively.

### Relationship between NO_3_^−^−N leaching and root nitrogen

NO_3_^−^−N leaching was clearly related to root TN (Fig. 5d). Treatment A, which had the lowest NO_3_^−^−N leaching, had the highest root TN. Treatment B showed the opposite relationship, whereas treatment C and the control showed intermediate results. Based on these findings, having a greater root volume improved the crop’s capacity to absorb NO_3_^−^−N.

### Nutrient budget and water balance

The TN in the soil at the end of the experiment was higher than that in the initial condition, indicating nitrogen absorption in the soil (Table 3a). Conversely, NO_3_^−^−N and NH_4_^+^−N in the soil decreased at the end of the experiment. This indicated that inorganic nitrogen in the soil was reduced by leaching or immobilization, whereas TN increased. Regarding inorganic nitrogen, treatment A showed the highest NO_3_^−^−N and NH_4_^+^−N, whereas treatment B had the second highest NO_3_^−^−N but the smallest NH_4_^+^−N (Table 3a). The ratio of NO_3_^−^−N and NH_4_^+^−N at depths of 0–30 cm with those at depths of 0–95 cm were 51%, 76%, 39%, and 59% and 87%, 89%, 85%, and 89% for the control and treatments A − C, respectively. This indicated that under treatment A, inorganic nitrogen tended to be in the shallow soil layer, whereas NO_3_^−^−N and NH_4_^+^−N under treatment B tended to move to deeper soil layers. The ratio of leached NO_3_^−^−N and NH_4_^+^−N and inorganic nitrogen in 0–95 cm depth were 95%, 71%, 114%, and 100% under the control and treatments A − C, respectively. This indicated that treatment A improved the absorption ability of NO_3_^−^−N and NH_4_^+^−N, mitigating leaching, while treatment B enhanced leaching. Overall, treatment C showed the same result as the control.

**Table 3:** Nutrient budget of TN and inorganic nitrogen and water balance.

	Control	A	B	C
(a) Nitrogen budget (mg)				
Fertilizer (NH_4_^+^−N)	1886			
Total crop (TN)	266	304	263	246
Aboveground	234	269	238	219
Root	31	35	25	28
Soil absorbed (TN)	3925	2805	3489	2120
Initial condition	24,249	24,210	24,014	24,210
Last condition	28,174	27,015	27,503	26,330
Inorganic nitrogen in soil (0–30 cm depth)	87	162	126	116
NO_3_^−^−N	17	31	12	19
NH_4_^+^−N	70	131	114	97
Inorganic nitrogen in soil (0–95 cm depth)	114	187	164	140
NO_3_^−^−N	34	40	30	32
NH_4_^+^−N	80	147	134	109
Leachate	33	29	37	33
NO_3_^−^−N	32	28	34	32
NH_4_^+^−N	1	1	3	1
(b) Water balance (mL)				
Irrigation	14,850			
Evapotranspiration	8740	10,194	9304	8930
Water in pipe	6638	6678	7630	6782
Drainage	7332	7120	7129	7234

Control: no biochar; A: surface application; B: plow layer application; C: subsurface application.

The amounts of water in the pipe were almost the same among the control and treatments A and C (Table 3b). Treatment B showed the highest amount of water because of higher *θ* at 80 cm depth, which represented 40 cm soil layer: the value might be overestimated. Evapotranspiration was the highest under treatment A followed by treatment B.

## Discussion

### Differences in the effects of biochar application depth in the absence and presence of the crop

#### Effects on soil water conditions

The differences between the treatments in *θ* were similar in our current findings (including the crop) and reported findings in no-crop condition^23^. The surface application of biochar (treatment A) caused a reduction in soil-water evaporation; thus, soil moisture in the deeper soil layers (20–35 cm depth) remained high; by contrast, plow layer biochar application (treatment B) caused soil-water evaporation to increase, thus, causing a reduction in soil moisture in the shallow soil layer (10 cm depth)^23^. The findings of the present study were similar (Fig. 3). Hamada et al.^23^ reported that subsurface biochar application (treatment C) induced a hydraulic barrier effect; this may explain the higher maximum *θ* and greater variability in *θ* at a depth of 10 cm observed in the present study. Here, the maximum *θ* in treatment C tended to be slightly higher (by 0.02 cm^3^ cm^− 3^) than in the control before February 14, 2022, potentially owing to the hydraulic barrier effect, although the increase was smaller than that previously reported^23^. However, after March 14, *θ* in treatment C became similar to that in the control. Presumably, the crop roots reached the biochar-amended soil layer close to or on that the day, affecting the soil pore structure, thus removing the effect of the hydraulic barrier. Nonetheless, this comparison reveals that the differences among the treatment in *θ* was affected predominantly by the biochar application depth.

#### Effects on nitrogen dynamics

In terms of NH_4_^+^−N and NO_3_^−^−N dynamics, our findings differ from those of Hamada et al.^23^. Dissolved NH_4_^+^−N content tended to be higher in the presence of the crop than without it (**Supplementary Fig. S3**), and soil NH_4_^+^−N content was smaller than that in Hamada et al.^23^ (**Supplementary Table S5**). Crops that receive NH_4_^+^−N fertilizer release H^+^ ions into the soil^38^. Here, soil NH_4_^+^−N was released via ion exchange, increasing the concentration of dissolved NH_4_^+^−N; this increase occurred after one fertilizer application (Fig. 2a), earlier than that previously reported^23^. For the control, Hamada et al.^23^ reported a higher dissolved NH_4_^+^−N concentration immediately after fertilizer application three (**Supplementary Fig. S3**). The soil in their study might have been saturated with NH_4_^+^−N by fertilizer application two; therefore, supplied NH_4_^+^−N by fertilizer application three directly dissolved in the soil solution. In the present study, root-induced release of NH_4_^+^−N did not lead to the soil becoming saturated with it.

Here, the concentration of NO_3_^−^−N in the soil solution tended to be lower than that in the previous study^23^ (**Supplementary Fig. S4**). Similarly, the soil NO_3_^−^−N content was lower here than previously reported (**Supplementary Table S5**) and lower than the initial value (Table 2). These differences reflect NO_3_^−^−N uptake by the crop. Here, treatments A and B exhibited different changes over time in NO_3_^−^−N at depths of 10 and 20 cm. NO_3_^−^−N is released slowly by biochar-amended soil^13^. Here, biochar addition to the surface soil layer caused the slow release of NO_3_^−^−N, thus, increasing dissolved NO_3_^−^−N content (Fig. 2b). NO_3_^−^−N was released faster from the soils in the other treatments (control and treatment C) than from the biochar-amended soil (treatments A and B), and the downward movement of NO_3_^−^−N might reduce the concentration of dissolved NO_3_^−^−N in the upper layer (Fig. 2b). However, this is not conclusive, as we performed sampling at relatively few depths and times. This should be evaluated in future simulation studies.

In summary, the nitrogen dynamics were altered by the presence of the crops, indicating that crops play a significant role in nitrogen dynamics.

#### Effects on drainage and nitrogen leaching

In the absence of the crop, drainage was significantly lower in treatment B than in the other treatments^23^: the increase in soil evaporation induced a reduction in drainage. Here, however, no significant differences in drainage were observed between the treatments (Fig. 5c). This indicates that the effect of treatment B (the reduction in drainage) was mitigated by crop presence. Because crop root growth was low in treatment B (Fig. 1b and c), water uptake by the crop was low, potentially masking the effect of biochar application in the plow layer. The smaller root growth in this treatment also led to an increase in NO_3_^−^−N leaching (Fig. 5d). Presumably, the lower root length density in the shallow soil layer (Fig. 1c) led to less efficient use of surface-supplied nitrogen, significantly increasing NH_4_^+^−N leaching (Fig. 5b). In Hamada et al.^23^, drainage increased in treatment A owing to reduced soil evaporation. By contrast, here, drainage tended to be lower in treatment A than in the other treatments (Fig. 5c). Treatment A exhibited the highest crop root growth (Fig. 1b and c) and water uptake. This high water uptake induced a decrease in drainage, which was not observed in the absence of crop. The higher water uptake induced by the better root growth also explains the reduction in NO_3_^−^−N leaching (Fig. 5d). Relative to the control, treatment C exhibited almost the same drainage and NO_3_^−^−N leaching (Fig. 5a and c). Here, even in the presence of the crop, subsurface application of biochar had a negligible effect, as observed in Hamada et al.^23^

These results indicate that the change in *θ* was affected primarily by biochar application depth. By contrast, nitrogen dynamics, drainage, and leaching were affected by the presence of the crops and differences in crop growth.

### Effects of soil-water stress on crop growth

Water balance estimation indicated that the evapotranspiration was higher in treatments A and B than in the others (Table 3b). Based on *h* condition and the effect of biochar application depth reported by Hamada et al.^23^, the high evaporation was owing to the increase in transpiration in treatment A but increase in evaporation in treatment B.

In treatment A, at all depths, *h* tended to remain in the no-stress range, indicating that treatment A generated less stress than the control (Fig. 4). This positively affected crop root growth (Fig. 1b), leading to increased root length density at all depths (Fig. 1c) and aboveground growth (Table 1; Fig. 1a), giving this treatment the highest NUE. In treatment B, dry stress occurred frequently, with *h* reaching the dry stress range before each irrigation (Fig. 4), preventing root growth in the surface soil layer and leading to a smaller root length density than in the control (Fig. 1c). After March 11, treatment B *h* at a depth of 10 cm was frequently less than − 1.0 × 10^4.2^ cm, and crop height was lower than that in the control (Table 1), indicating an adverse effect of dry stress on crop growth. Although the soil physical properties did not differ significantly among the treatments, wet stress occurred in the bottom soil layer in treatment B, leading to a low root length density (Fig. 1c). However, we were unable to identify the reason for this wet stress. In general, soil physical properties can be determined via laboratory experiments; nonetheless, a 100 mL sample does not always represent the soil layer (e.g. Nakamura et al.^39^), and parameter calibration using a simulation model is effective for evaluating soil-water movement^40^. Therefore, in future studies, calibration should be conducted to explain the occurrence of wet stress. In treatment C, dry stress occurred frequently in the shallow soil layers. The lower root length density in the shallow soil layer indicated that dry stress prevented root growth in treatment C. Interestingly, dry stress at a depth of 10 cm occurred less frequently in treatment C than in treatment B; however, aboveground growth tended to be smaller in treatment C than in treatment B. This implies that another factor affects root growth. By contrast, the dissolved NO_3_^−^−N content (Fig. 2b) tended to be high in treatment B and low in treatment C, potentially affecting crop growth. This aspect should be evaluated in future studies.

These findings indicate that soil-water stress varied with biochar application depth, affecting root growth and consequently aboveground growth.

### Effect of biochar application depth on NO_3_^−^−N and NH_4_^+^−N leaching under crop present condition

The nutrient budget for TN revealed that TN in soil increased and the degree was higher in the control and treatment B than in the other two treatments (Table 3a). However, regarding NO_3_^−^−N and NH_4_^+^−N, the trends were different. The estimation of NO_3_^−^−N and NH_4_^+^−N in soil indicated that their absorption abilities improved in treatment A, and more than 75% remained at 0–30 cm depth, mitigating leaching. Moreover, crop nitrogen intake was considered the highest in treatment A, further reducing leaching. By contrast, in treatment B, NO_3_^−^−N and NH_4_^+^−N moved to the deeper soil layers (30–95 cm) rather than remaining at 0–30 cm depths, increasing NO_3_^−^−N and NH_4_^+^−N leaching (Fig. 5a and b). The crop growth condition promoted the adverse effect on the leaching. The amounts of NO_3_^−^−N and NH_4_^+^−N were higher in treatment C than in the control at 0–30 cm; however, this had little effect on leaching.

Our results showed that biochar application depth affected the absorption ability of NO_3_^−^−N and NH_4_^+^−N in soil and soil-water stress conditions, consequently affecting crop growth, which could enhance the mitigation of leaching. Biochar application at 0–5 cm depths showed the maximum effect on the mitigation of leaching and improvement of crop growth.

We note some limitations of this study. Soil sampling points were scarce; therefore, a more intense sampling or simulation is necessary to comprehensively evaluate NO_3_^−^−N and NH_4_^+^−N content in soil. Moreover, we evaluated root growth using only two replicates and, thus, could not apply statistical tests. Moreover, biochar acts as a nitrification inhibitor^41,42^. To comprehensively understand its effects, the effects of biochar application depth on nitrogen leaching and nitrogen loss as gas (e.g., as N_2_O) should be examined.

## Conclusion

A pipe experiment was conducted to evaluate the interactions between biochar application depth and crop growth and their effect on nitrogen leaching and understand the mechanism driving the interaction. Biochar application depth affected NO_3_^−^−N and NH_4_^+^−N absorption ability. Moreover, the application depth changed soil-water stress conditions, thus, affecting crop growth. After surface biochar application, NO_3_^−^−N and NH_4_^+^−N absorption improved, and they tended to remain at 0–30 cm depths. Soil-water stress tended to be low owing to the reduction in soil-water evaporation, improving crop growth. These two changes reduced NO_3_^−^−N leaching. After plow layer biochar application, NO_3_^−^−N and NH_4_^+^−N absorption worsened, and they tended to move to 30–95 cm depths. Moreover, the resulting increase in soil-water evaporation caused dry stress in the shallow soil layer. This prevented crop root growth in this soil layer, causing less aboveground crop growth and nitrogen uptake. These changes increased NO_3_^−^−N and NH_4_^+^−N leaching. Finally, subsurface biochar application had little effect relative to the no biochar application. To comprehensively understand the effect of biochar application depth, future studies should evaluate the distribution of NO_3_^−^−N and NH_4_^+^−N in soil by dense soil sampling or simulation. Nitrogen loss as gas should also be investigated to better understand this effect. Based on our findings, we believe that selecting the appropriate biochar application depth is likely to promote agriculture sustainability.

## Electronic supplementary material

Below is the link to the electronic supplementary material.

Supplementary Material 1

## Data Availability

Data is provided within the manuscript or supplementary information files.
