# Supplementary material for: Pipe experiment elucidates biochar application depth affects nitrogen leaching under crop present condition
Source: Sci Rep. 2024 Oct 1;14:22823. doi: 10.1038/s41598-024-73621-3 (PMC11445262; doi:10.1038/s41598-024-73621-3)
Supplement: Supplementary file 1 — Supplementary Material 1 [file 41598_2024_73621_MOESM1_ESM.docx]

**Supplementary information for**

**Biochar application depth affects crop growth and nitrogen leaching**

Kosuke Hamada ^a, *^, Satoshi Nakamura ^b^, Daichi Kuniyoshi ^a^

^a^ Tropical Agriculture Research Front, Japan International Research Center for Agricultural Sciences, Ishigaki, Japan

^b^ Crop, Livestock and Environmental Division, Japan International Research Center for Agricultural Sciences, Tsukuba, Japan

^*^ Corresponding author, E-mail: [hamadak302@affrc.go.jp](mailto:hamadak302@affrc.go.jp)

**Table S1** Chemical and physical properties of the soils

|  | pH | EC | Bray1–P | Bray2–P | NO_3_^−^−N | NH_4_^+^−N | T–N | T–C |
| --- | --- | --- | --- | --- | --- | --- | --- | --- |
|  | (H_2_O) | mS m^–1^ | mg kg^–1^ | | | | g kg^–1^ | |
| Control | 5.25 | 4.28 | 4.20 | 25.81 | 1.29 | 5.81 | 0.65 | 3.59 |
| Coarse | 5.21 | 4.20 | 5.04 | 31.45 | 1.23 | 7.25 | 0.63 | 3.63 |
| Dense | 5.21 | 4.42 | 4.69 | 35.23 | 1.27 | 6.80 | 0.63 | 3.88 |

|  | Exchangeable Cations | | | |  |  |  |  |  |
| --- | --- | --- | --- | --- | --- | --- | --- | --- | --- |
|  | Ca | K | Mg | Na | CEC | Sand | Silt | Clay | Texture |
|  | cmolc kg^–1^ | | | | cmolc kg^–1^ | % | | |  |
| Control | 2.05 | 0.13 | 0.83 | 0.13 | 8.52 | 41.0 | 10.3 | 41.0 | Heavy clay |
| Coarse | 2.22 | 0.14 | 0.85 | 0.14 | 8.89 | 35.9 | 15.6 | 48.3 | Heavy clay |
| Dense | 2.13 | 0.15 | 0.87 | 0.14 | 9.22 | 28.3 | 14.4 | 57.3 | Heavy clay |

Control: no biochar application, Coarse: biochar amendment layer under treatment B (plow layer application), Dense: biochar amendment layer under treatments A and C (surface and subsurface application)

**Table S2** Chemical properties of biochar

| pH | EC | Ash content | T–N | T–C | C/N | Total | | | | | Water soluble | | | | |
| --- | --- | --- | --- | --- | --- | --- | --- | --- | --- | --- | --- | --- | --- | --- | --- |
|  |  |  |  |  |  | Ca | K | Mg | Na | P | Ca | K | Mg | Na | P |
| 1:10 | mS m^–1^ | % | % | | | g kg^–1^ | | | | | g kg^–1^ | | | | |
| 7.69 | 20.55 | 94.7 | 0.05 | 3.66 | 72.1 | 2.70 | 0.35 | 0.58 | 0.35 | 1.14 | 0.07 | 0.17 | 0.07 | 0.07 | 0.12 |

**Table S3** Saturated soil hydraulic conductivity

| Depth | Control | A | B | C |
| --- | --- | --- | --- | --- |
| 0−5 | 4.5×10^−2^ | 2.0×10^−2^ | 2.0×10^−2^ | 3.4×10^−2^ |
| 25−30 | 2.8×10^−2^ | 2.8×10^−2^ | 3.6×10^−2^ | 2.7×10^−2^ |
| 50−55 | 3.1×10^−2^ | 2.1×10^−2^ | 2.9×10^−2^ | 1.4×10^−2^ |

Unit: cm s^−1­­­^. Control: no biochar; A: surface application; B: plow layer application; C: subsurface application. The average values of three replicates are displayed

**Table S4** van Genuchten parameters for the measured soil water retention curves

|  | θ_res_  (cm^3^ cm^−3^) | θ_sat_  (cm^3^ cm^−3^) | α  (cm^−1^) | n  (−) |
| --- | --- | --- | --- | --- |
| Control 5 cm | 0.15 | 0.58 | 0.08 | 1.74 |
| A 5 cm | 0.14 | 0.60 | 0.07 | 1.70 |
| B 5 cm | 0.15 | 0.54 | 0.15 | 1.50 |
| C 5 cm | 0.15 | 0.53 | 0.10 | 1.50 |
| 30 cm | 0.15 | 0.52 | 0.09 | 1.60 |
| 55 cm | 0.15 | 0.43 | 0.70 | 1.70 |

Control: no biochar; A: surface application; B: plow layer application; C: subsurface application. *θ_res_*: residual volumetric water content; *θ_sat_*: saturated volumetric water content; *α* and *n*: empirical shape factors.

**Table S5** NO_3_^−^−N and NH_4_^+^−N contents in the soil with/without a crop

| (a) NO_3_^−^−N (mg kg^–1^) | | | | | | | | |
| --- | --- | --- | --- | --- | --- | --- | --- | --- |
| Depth  (cm) | Control | | A | | B | | C | |
|  | No crop | With a crop | No crop | With a crop | No crop | With a crop | No crop | With a crop |
| 5 | 93.3 | 2.9 | 76.2 | 3.5 | 79.3 | 1.4 | 76.5 | 2.7 |
| 30 | 13.4 | 0.9 | 12.9 | 0.7 | 8.6 | 0.6 | 10.4 | 0.7 |
| 55 | 7.4 | 0.6 | 7.8 | 0.6 | 8.0 | 0.7 | 7.9 | 0.6 |
|  |  |  |  |  |  |  |  |  |
| (b) NH_4_^+^−N (mg kg^–1^) | | | | | | | | |
| Depth  (cm) | Control | | A | | B | | C | |
|  | No crop | With a crop | No crop | With a crop | No crop | With a crop | No crop | With a crop |
| 5 | 224.8 | 22.0 | 131.2 | 16.7 | 159.4 | 22.0 | 177.8 | 26.2 |
| 30 | 14.4 | 1.3 | 11.6 | 1.6 | 15.9 | 2.8 | 15.6 | 1.0 |
| 55 | 13.0 | 1.1 | 13.3 | 1.7 | 15.9 | 1.7 | 14.9 | 1.3 |

Control: no biochar; A: surface application; B: plow layer application; C: subsurface application. Values represent the means of three replicates. The result with no crop was from Hamada et al. (2023)

**
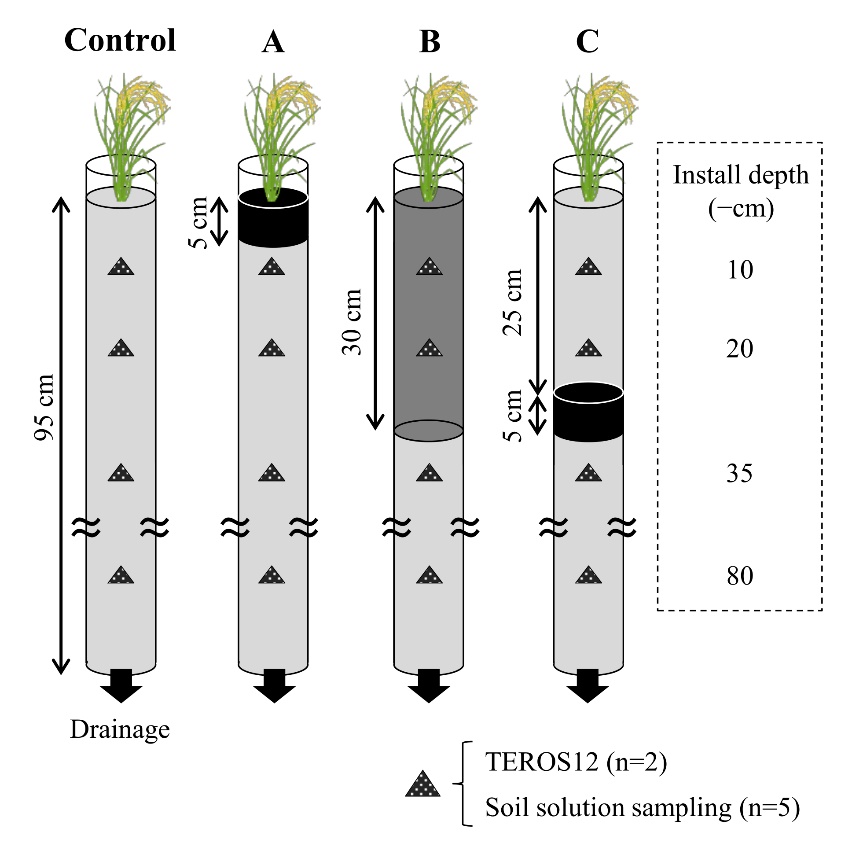
**

**Fig. S1** Schematic of the experimental pipes

Control: no biochar; A: surface application; B: plow layer application; C: subsurface application

**
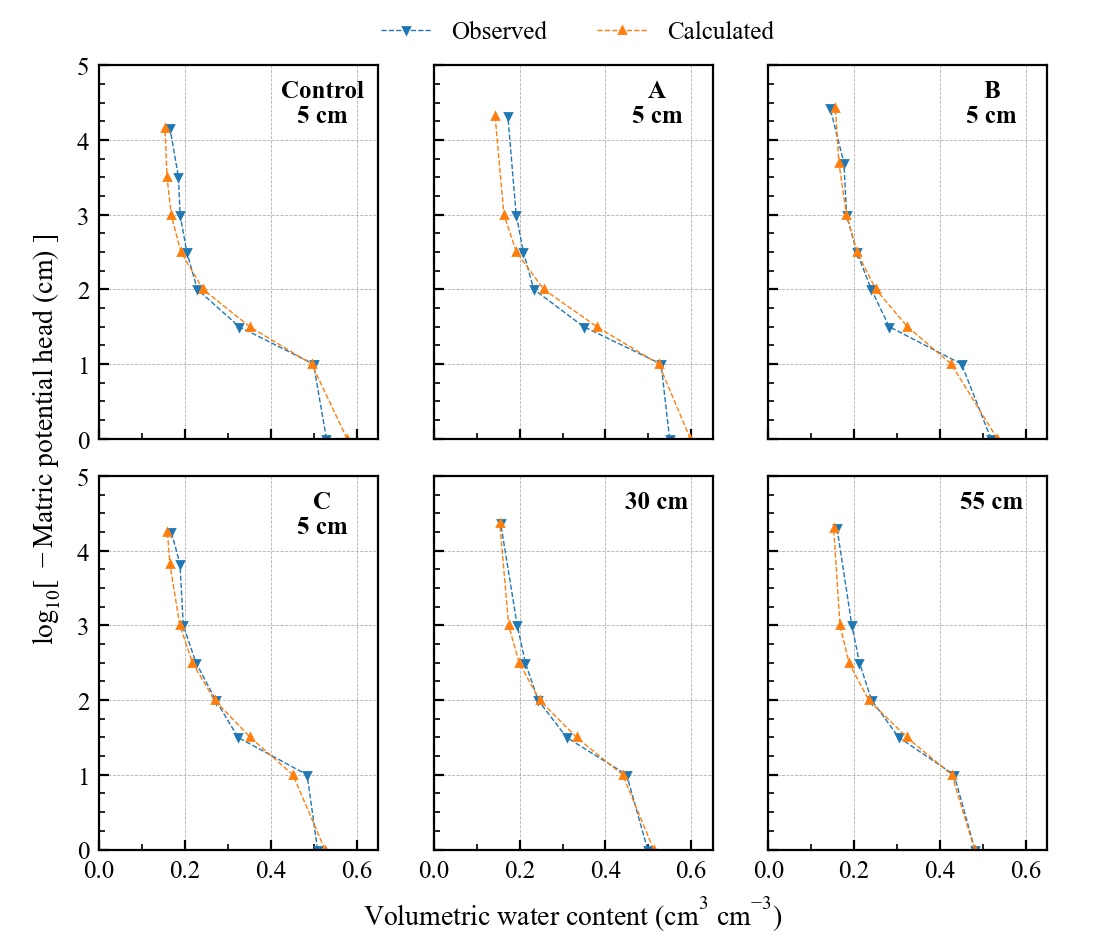
**

**Fig. S2** Soil water retention curves

Control: no biochar; A: surface application; B: plow layer application; C: subsurface application. Calculated curves were obtained from van Genuchten equation


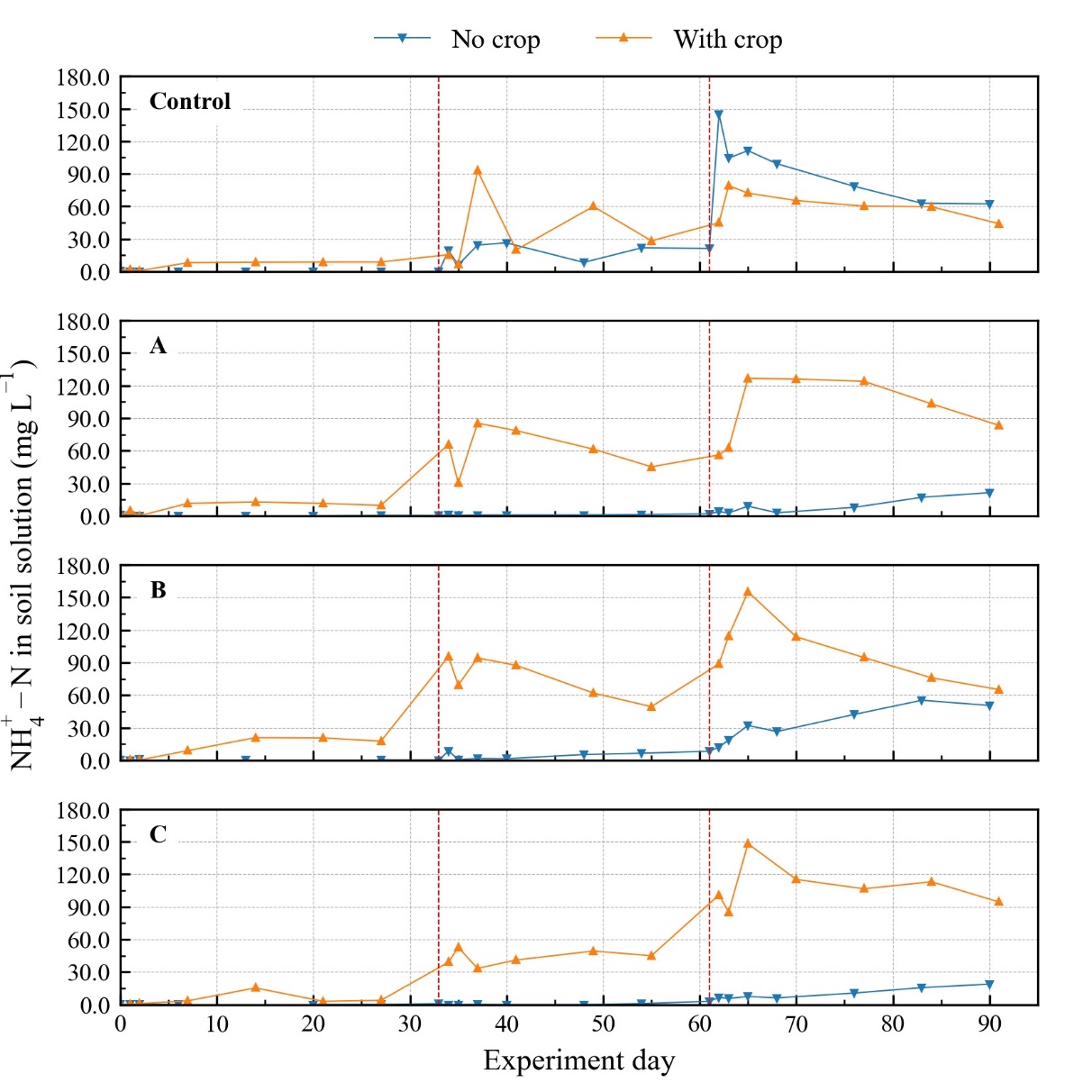


**Fig. S3** Temporal changes in the concentration of dissolved NH_4_^+^−N at a depth of 10 cm with/without a crop

Control: no biochar; A: surface application; B: plow layer application; C: subsurface application. The vertical red dashed lines indicate fertilizer application. The average values of three replicates are shown. The result with no crop was from Hamada et al. (2023)


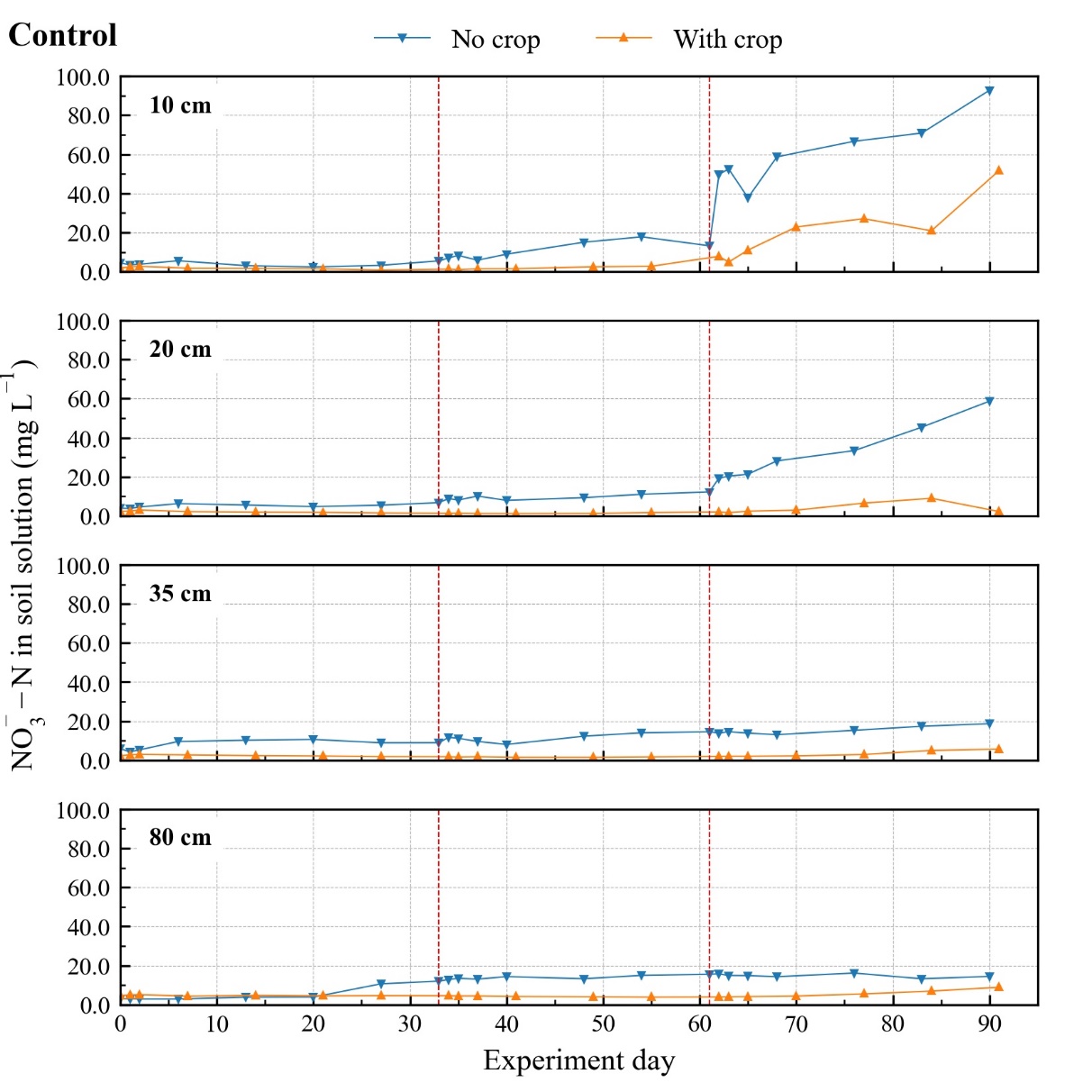


**Fig. S4 (**a) Temporal changes in the concentration of dissolved NO_3_^−^−N at each depth with/without a crop

Control: no biochar. The vertical red dashed lines indicate fertilizer application. The average values of three replicates are shown. The result with no crop was from Hamada et al. (2023)


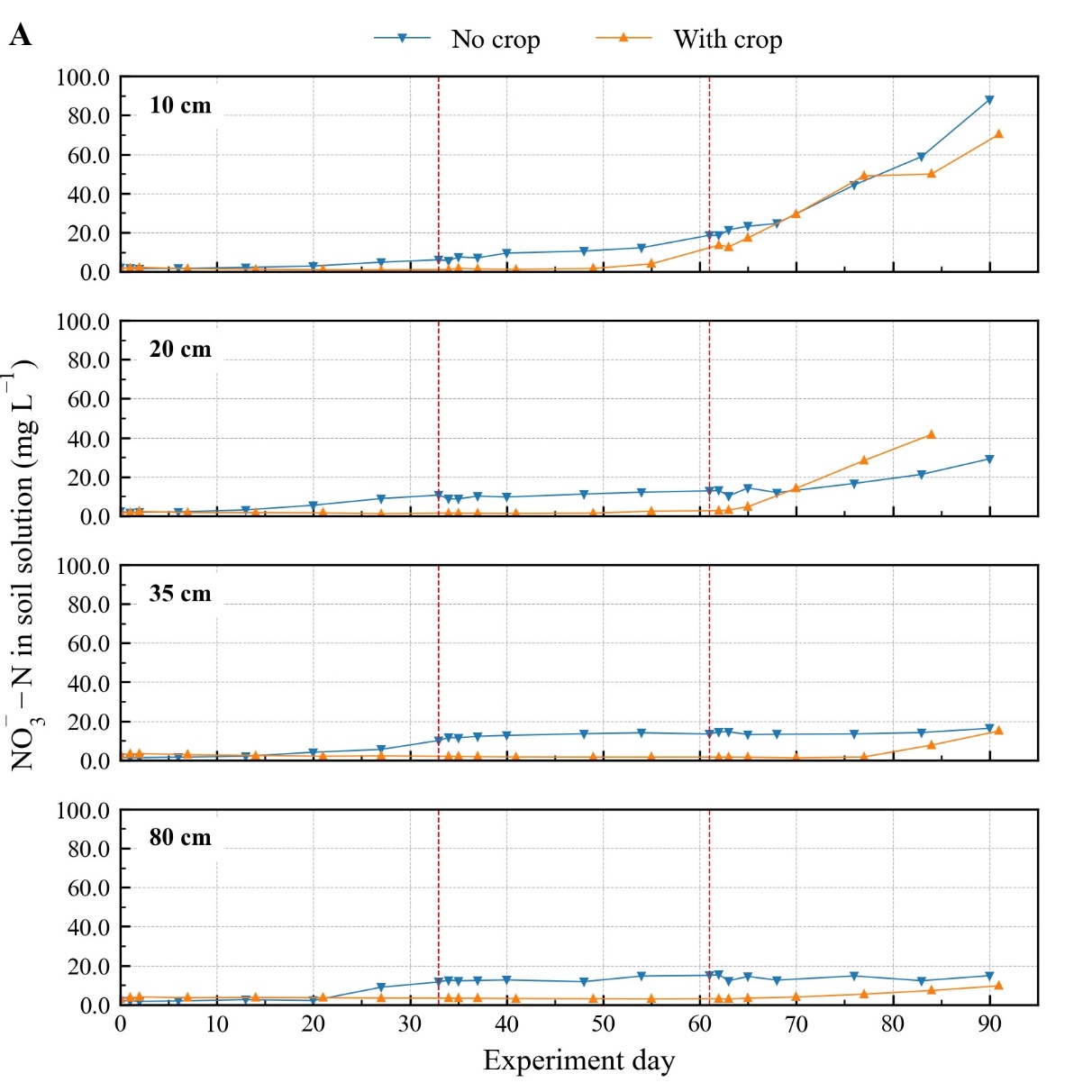


**Fig. S4 (**b) Temporal changes in the concentration of dissolved NO_3_^−^−N at each depth with/without a crop

A: surface application. The vertical red dashed lines indicate fertilizer application. The average values of three replicates are shown. The result with no crop was from Hamada et al. (2023)


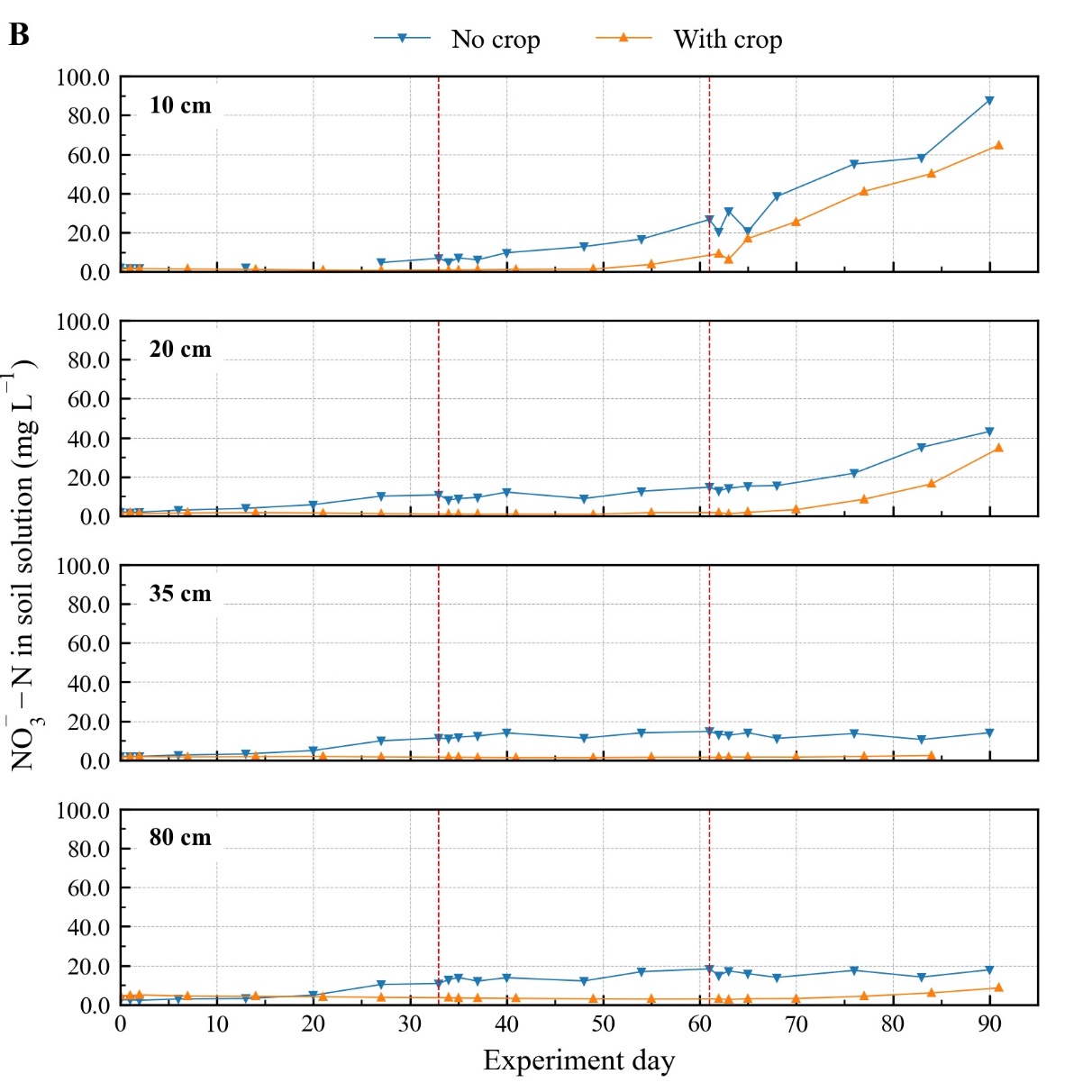


**Fig. S4 (**c) Temporal changes in the concentration of dissolved NO_3_^−^−N at each depth with/without a crop

B: plow layer application. The vertical red dashed lines indicate fertilizer application. The average values of three replicates are shown. The result with no crop was from Hamada et al. (2023)


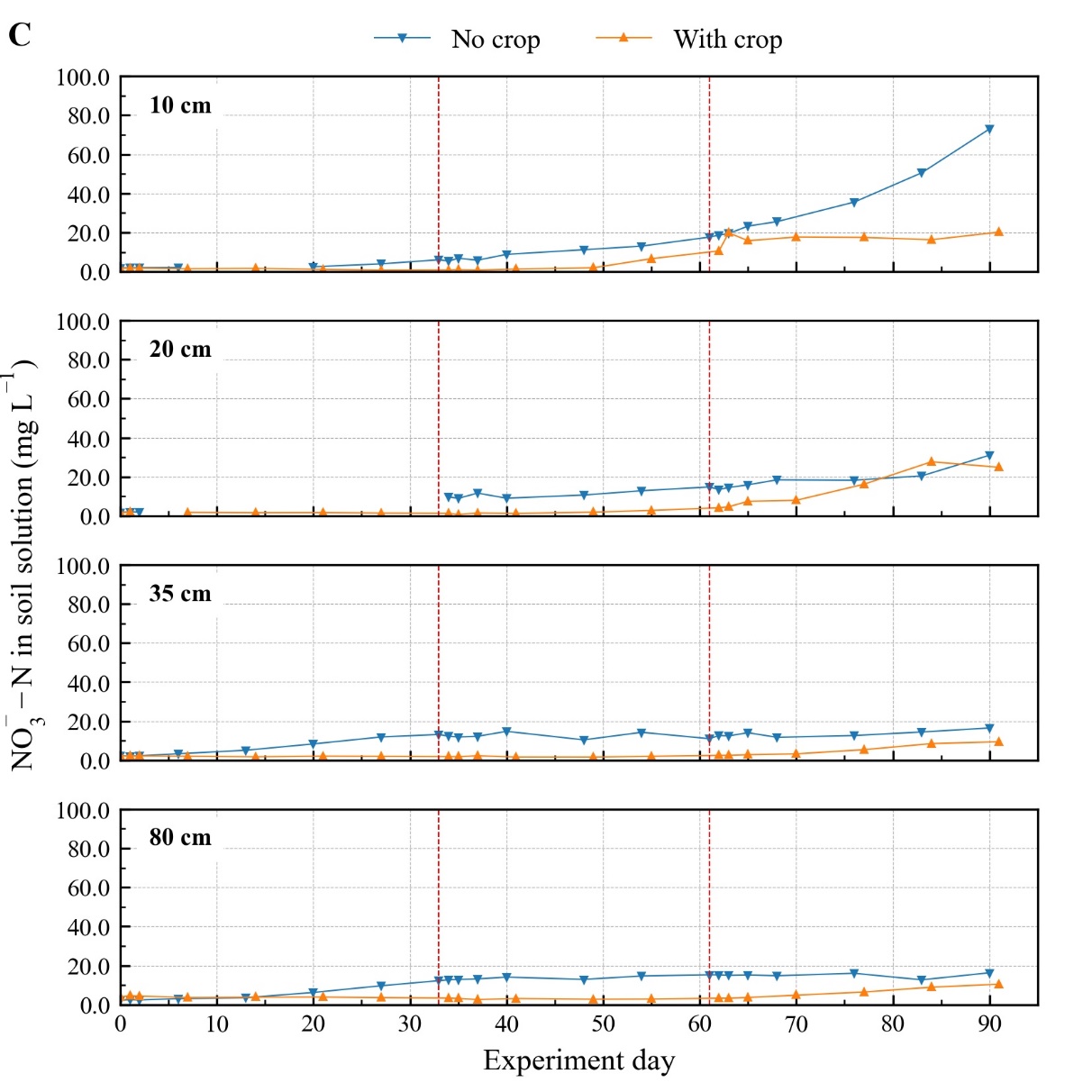


**Fig. S4 (**d) Temporal changes in the concentration of dissolved NO_3_^−^−N at each depth with/without a crop

C: subsurface application. The vertical red dashed lines indicate fertilizer application. The average values of three replicates are shown. The result with no crop was from Hamada et al. (2023)
